# Circular economy reinforcement through molecular fabrication of textile wastes with microbial synthesized ZnO nanoparticles to have multifunctional properties

**DOI:** 10.1038/s41598-024-66430-1

**Published:** 2024-07-19

**Authors:** Osama M. Darwesh, Ibrahim A. Matter, Naser G. Al-Balakocy, Mohamed I. Abo-Alkasem

**Affiliations:** 1 Agricultural Microbiology Department, National Research Centre, 33 EL-Buhouth St., Dokki, Cairo 12622 Egypt; 2 Protenic and Manmade Fibers Department, National Research Centre, Dokki, Cairo 12622 Egypt; 3 Chemistry of Natural and Microbial Products Department, National Research Centre, Dokki, Cairo 12622 Egypt

**Keywords:** Recycle PET/C fibrous waste, Enzymatic treatment, Actino-synthesized ZnO-NPs, Antimicrobial, Ultraviolet protection, Biological techniques, Biotechnology, Microbiology

## Abstract

The fibrous wastes generated from the mills of textile production can be recycled and converted into high add-values products to be implemented in several applications. The current study aimed to employ commercial free cellulase enzyme to partially hydrolyze (activate) the polyester cotton blended (PET/C) fibrous wastes by creation functional groups such as OH and COOH on their surfaces. The activated fibrous wastes were then modified by coating with ZnO nanoparticles (ZnO-NPs) biosynthesized by actinobacterial cultures free supernatant. The isolate was identified as *Streptomyces pseudogriseolus* with accession number of OR574241. The conditions that influence the actino-synthesis of ZnO-NPs were optimized and the product was characterized using spectroscopic vision, FTIR, XRD, TEM and SEM. The characteristic ZnO peaks were obviously observed by EDX analysis with 0.38 and 0.75% (wt%), respectively. TEM analyses proved the nanoscale of ZnO-NPs (5–15 nm) which was followed by cytotoxic evaluation for the produced NPs. Fortunately, the tested actino-ZnO-NPs didn’t have any cytotoxicity against human normal fibroblast cell line (BJ1), which means that the product can be safely used in a direct-contact with human skin. The treated PET/C blended waste fabrics coated with ZnO-NPs showed high antimicrobial activity and ultraviolet protection values after functionalization by cellulase. EDX analysis demonstrates the presence of Zn peaks on the coated fabrics compared with their absence in blank and control samples, while SEM images showed the formation of a thin layer of ZnO-NPs on the fabric surface. The obtained smart textile can be applied several needed sectors.

## Introduction

Due to fast fashion and the world’s rising population, the textile sector has shown a sharp growth pattern in recent years, producing 92 million tons of textile waste globally each year. A 85% of this waste is disposed of in landfills, which pollutes the environment^1^. Scientists face difficulty in trying to recycle these massive amounts of waste without creating another sort of pollution from harsh chemicals^2^. Thus, the fibrous wastes produced by textile manufacturing facilities can be recycled and transformed into goods with high added values for use in various applications. One of these valuable applications is to be used as wound dressing materials that are mainly made up of natural polymers because they are biocompatible and biodegradable materials^3–5^.

The nature, chemical structure, and moisture content of textile materials promote the growth of microorganisms which have an adverse effect on the properties of these materials such as loss in mechanical properties, changes in textile color, and emission of unfavorable odors^6^. So, the demand for developing textile materials with multifunctional properties increases. A lot of efforts have been spent recently by researchers to establish textile products with antimicrobial activity, consequently, using of antimicrobial agents such as metal oxide nanoparticles with unusual mechanisms of action may provide a glimmer of hope in overwhelming multi-drug-resistant microorganisms^7^.

Fabrication of textile materials to possess antimicrobial activity is controlled by several factors such as the type of antimicrobial agent as well as fiber composition, type, and texture of fiber surface. Hence, the way of antimicrobial agent may interact with the fibrous material varies between direct contact and diffusion. For instance, Cotton fabrics have complex structures made up of cellulose molecules, which are polymers created from D-glucose monomers through β-(1,4) glycosidic bonds. These polymers are produced by cellulose synthase complexes, forming tiny fibrils about 1.5–3.5 nm wide. These fibrils can aggregate to create larger bundles called cellulose nanofibers, which eventually form microfibers with diameters of several micrometers. However, most methods for creating antiviral and antibacterial fabrics involve simply adding these agents to the cotton material, without considering the fabric's molecular structures^6^.

On the other hand, one of the fast-growing technologies in textile manufacturing is using enzymes as a biocatalyst. Application of enzymes is an eco-friendly technology because they are considered a natural alternative to harsh chemicals and due to the remarkable reduction in power consumption and their reusability make them the most proper cost-effective methods for many applications. Cellulases are enzymes that break down cellulose into smaller sugars, eventually converting it to glucose. Three main types of cellulases work together to break down cellulose: endoglucanases, cellobiohydrolases, and β-4-glucosidase. Accordingly, during the current study, cellulase enzymes were used to activate the surface of polyester cotton blend (PET/C) and polyester (PET) at the molecular level by introducing polar groups such as OH and COOH groups on the material-based polyester surface which facilitates the binding of nanoparticles with fiber molecules which secure the long-term stability of the antimicrobial activity for the fabricated material^10^. The nanoparticles eco-friendly and sustainable production without use toxic solvents or hazardous chemicals, has gained attention in recent years within biological processes especial use microorganisms.

On the other hand, the choice of the proper NPs to be used as an antibacterial agent is another devastating step to selecting the compounds that don’t exert any harmful impact on human health or even induce toxic reactions that may cause environmental issues^11,12^. ZnO-NPs have been reported by the US Food and Drug Administration as safe substances (GRAS) due to their biosafety, biocompatibility, and lack of toxicity even after daily use of the ZnO-NPs^13^. Therefore, the current work aimed to produce smart textiles from polyester/cotton blended wastes after activation by cellulase enzyme to accept loading actino-synthesized ZnO-NPs onto their activated surface.

## Results and discussion

### Biological activation of fibrous waste

To achieve long-term and durable multifunctional properties during the recycling process of textile wastes, fabrication was conducted through three different steps: cleaning, activation, and molecular modification. However, the enzymatic activation works on improving the physical and chemical properties of the textile material, but on the other hand, a minor decrease in the material weight is also noticed, Table 1 summarizes the changes in fabric weight in addition to the noteworthy increase in the carboxylic content after enzymatic treatment which facilitate the binding of ZnO-NPs on the fabric surface. The direct result of partial enzymatic hydrolysis of the cellulose fibers, particularly on the fabric surface and amorphous regions, could be the loss of material weight. This process yields soluble molecules like glucose and short-chain oligomers^14^. The loss in weight (4.5%) may also be attributed to the higher specific activity of cellulases concerning cotton fibers than polyester resulting in the dissociation of cellulosic bonds.

**Table 1:** Influence of the cellulase treatment on the weight loss, carboxylic content, and actino-synthesized ZnO-NPs loaded on PET/C blended fabric wastes.

Fabrics	Weight loss%	Carboxylic content (meq/100 g fabric)	Zn content (Atomic%) estimated by EDX
PET/C	0.0	8.10	0.0
PET/C/E	4.5	36.5	0.0
PET/C/E/ZnO-NPs			0.75

PET/C, Polyester/Cotton; E, cellulases enzyme; ZnO-NPs, actino-synthesized ZnO-NPs.

### Actinobacterial production of ZnO-NPs

**Figure 1:**
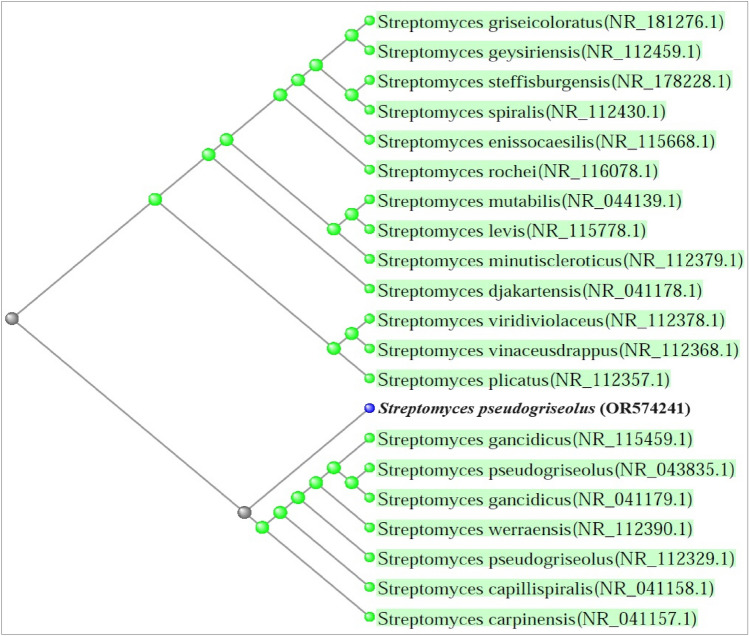
The created phylogenetic tree constructed from the 16S rDNA sequence of NRC-MO23 actinobacterial isolate and their related reference Genbank strains.

A total of 64 morphologically distinct isolates were isolated and screened for their ability to resist Zn ions supplemented in the growth medium and the results showed that eight isolates were found to have the ability to grow in the presence of Zn ions. These isolates coded as NRC-MO10, NRC-MO19, NRC-MO21, NRC-MO23, NRC-MO31, NRC-MO40, NRC-MO52 and NRC-MO62. Morphological differences between these isolates were recorded in Tables S1–S8, and then screened for their ability to form Zn nanostructure. Different absorption between the eight isolates was noted. Based on that, an isolate coded with NRC-MO23 was selected as the most potent one to produce large amounts of ZnO-NPs. The selected actinobacterial isolate was subjected to an identification step. The morphological and cultural characteristics as noted in Table S4 were led to identify this isolate as *Streptomyces pseudogriseolus.* The molecular identification of this isolate was done to confirm the classical identification technique. After DNA isolation and amplification of 16s rDNA, the sequence data led to identify this isolate as *Streptomyces pseudogriseolus* with accession number OR574241 (Fig. 1).

### Characterization of the actino-synthesized ZnO-NPs

The produced ZnO-NPs were characterized using different techniques such as FTIR, XRD, and SEM–EDX. The morphology and topography were studied using SEM, while, the size and formation shape were considered by TEM.

### The visual observation

The first primitive inspection method to determine the formation of the ZnO nanostructure is the change in reaction color into a yellowish or milky color (Fig. S1). This change in the reaction color was also reported by several researchers for instance; Vidya and Arulpandi^15^ observed the formation of white cloudy haziness in the solution which eventually settled at the bottom of the flask as the nanoparticles were formed by yeast culture of *Pichia fermentans.* Also, Kavitha and his co-worker^16^ reported similar observations during the preparation of ZnO-NPs using terpenoid fractions of *Andrographis paniculate* leaves as the color changed from transparent to cloudy white. Similarly, Radwan et al.,^17^ found that the color of mixtures (mixing the Zn ions with the microalgal metabolites solution) was changed and precipitates of ZnO-NPs formed.

### UV–visible spectrometer

**Figure 2:**
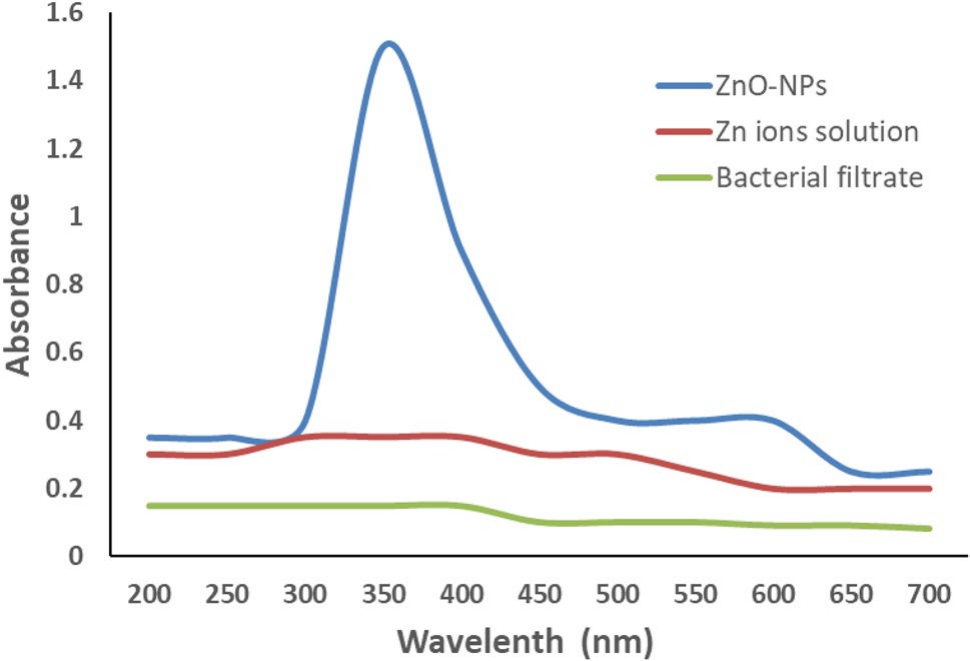
UV–visible absorption spectra of ZnO-NPs suspension.

UV–Vis spectroscopic analysis was made to confirm the formation of ZnO-NPs. The results illustrated in Fig. 2 showed that Lambda max (λmax) was observed at 350 nm, which attributed to the intrinsic bandgap of ZnO absorption^18^.

### Estimation of particle size for the actino-synthesized ZnO-NPs

**Figure 3:**
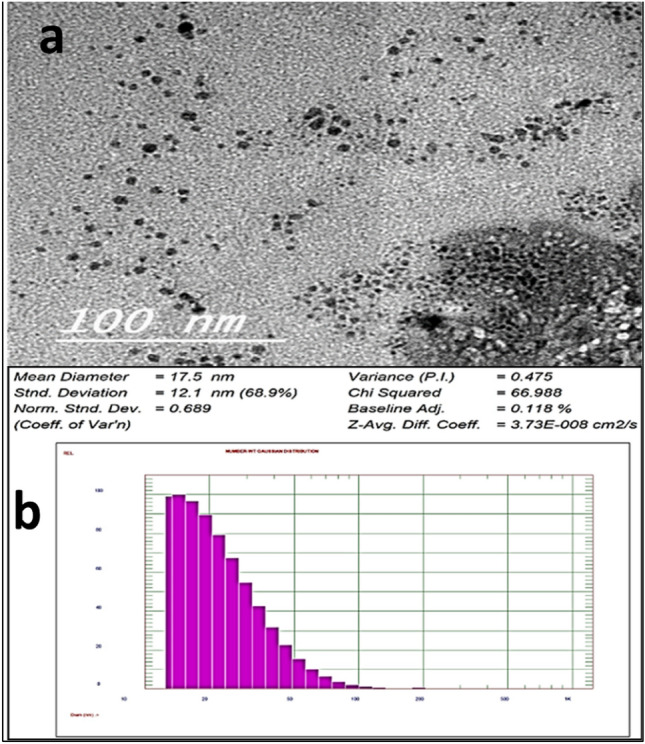
Examination of the size and morphological shape of bio-synthesized ZnO-NPs by TEM (**a**) and DLS analysis (**b**).

TEM imaging was used to to give insight about size and morphology. The TEM micrographs of the actino-synthesized ZnO-NPs show differences in agglomerate size distribution (Fig. 3). The ZnO-NPs size reached 5–15 nm. The small particle size supports the beneficial applications of zinc oxide nanoparticles.

### Chemical Composition (FTIR)

**Figure 4:**
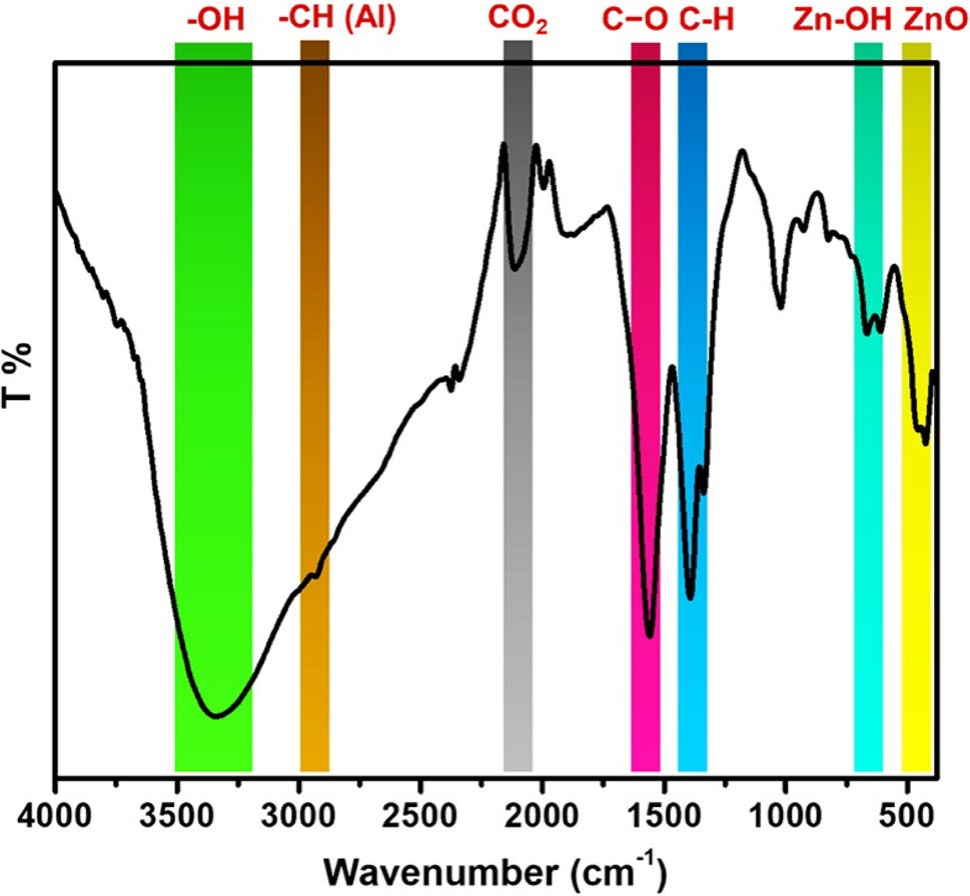
FTIR spectrum of actinobacterial-ZnO-NPs.

The likely biomolecules in charge of ZnO-NPs reduction, capping, and efficient stabilization were identified using FTIR. FTIR analysis for the biosynthesized ZnO-NPs was conducted to give insights into the type of functional groups responsible for the transformation of simple inorganic Zn salts to elemental forms. As a result, the formed ZnO-NPs can act as stabilizing, reducing, and capping agents. FTIR measurements delivered probable insight into the surface chemistry of actino-synthesized ZnO-NPs by identifying the functional groups of microbial biomolecules attached with them, which produced their biosynthesis and stabilization. The FTIR spectrum of ZnO nano-powder produced in the bacterial suspension is depicted in Fig. 4. As shown, several absorption bands are pronounced in the range between 4000 and 400 cm^−1^. Particularly, a broad peak can be observed at 3340 cm^−1^ corresponds to the –OH stretching^19^. It is worth mentioning that the surface of ZnO is rich in hydroxyl groups, via the adsorption of water molecules, that could be further interacted with other substrates^20^. Therefore, the Zn-OH bond was noticed at ~ 615 cm^−1^ ^19^. Additionally, weak peaks were detected at 2928 and 2846 cm^−1^ which refers to stretching of aliphatic –CH group^20^, while other peaks assigned at ~ 1400 and ~ 1560 cm^−1^ revealed C–O symmetric and asymmetric stretching vibrations, respectively^20^. Indeed, these bands might be attributed to the residual of the reductants released by bacteria during the precipitation of ZnO nanoparticles. These secretions not only act as reducing agents for zinc acetate substrate but also as capping agents for the obtained particles. As the FTIR analysis is usually performed under an ambient atmosphere, carbon dioxide gas can be detected by the device. Therefore, the peak assigned shifted at ~ 2110 cm^−1^ which might be due to CO_2_^19^. On the other hand, FTIR precisely confirmed the formation of the ZnO structure as the band of the Zn–O bond was strongly marked at ~ 425 closely in agreement with the state of art^20^. No doubt, it has been previously reported that ZnO-NPs can strongly interact with other substrates including cellulose on the molecular level thanks to its surface functionality^21^.

### XRD spectroscopy

**Figure 5:**
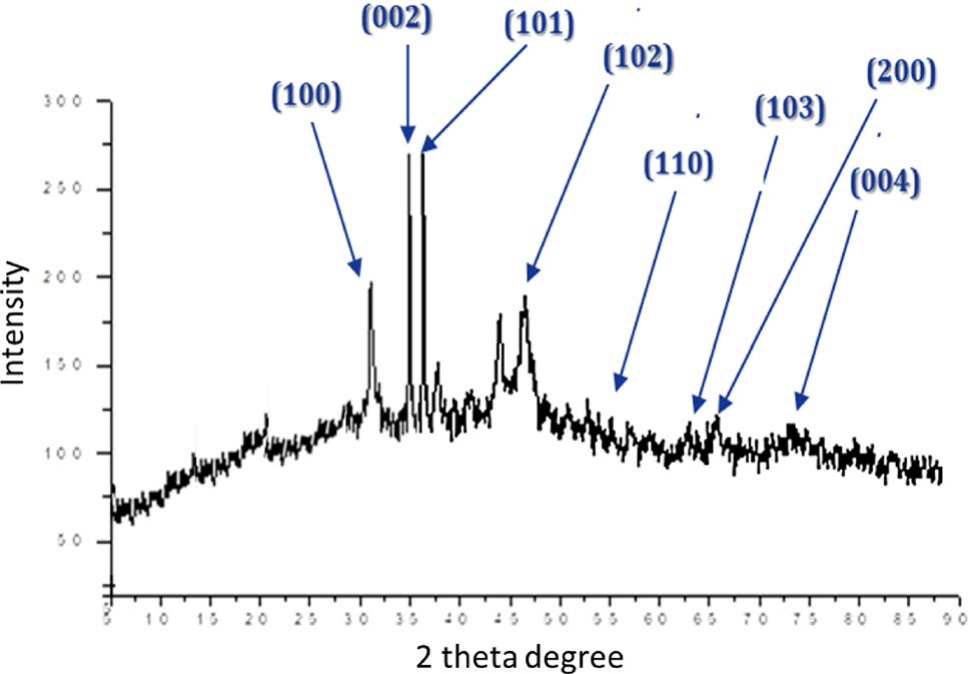
XRD pattern of bacterial synthesized ZnO-NPs.

XRD diffractogram of the actino-synthesized ZnO-NPs presented in Fig. 5 revealed the diffraction of the peak positions with 2θ values of 31.6°, 34.46°, 36.26°, 47.5°, 55.54°, 62.5°, 65° and 72.4° are indexed as (100), (002), (101), (102), (110) (103), (200) and (004) planes, there was no detection of extra diffracted peaks of other phases which indicated the phase purity of ZnO nanopowder. These results matched the results of the standard ZnO bulk (Joint Committee on Power Diffraction Standards, JCPDF-36-1451). And agree with the data reported previously^21,22^.

### SEM and EDX investigations

**Figure 6:**
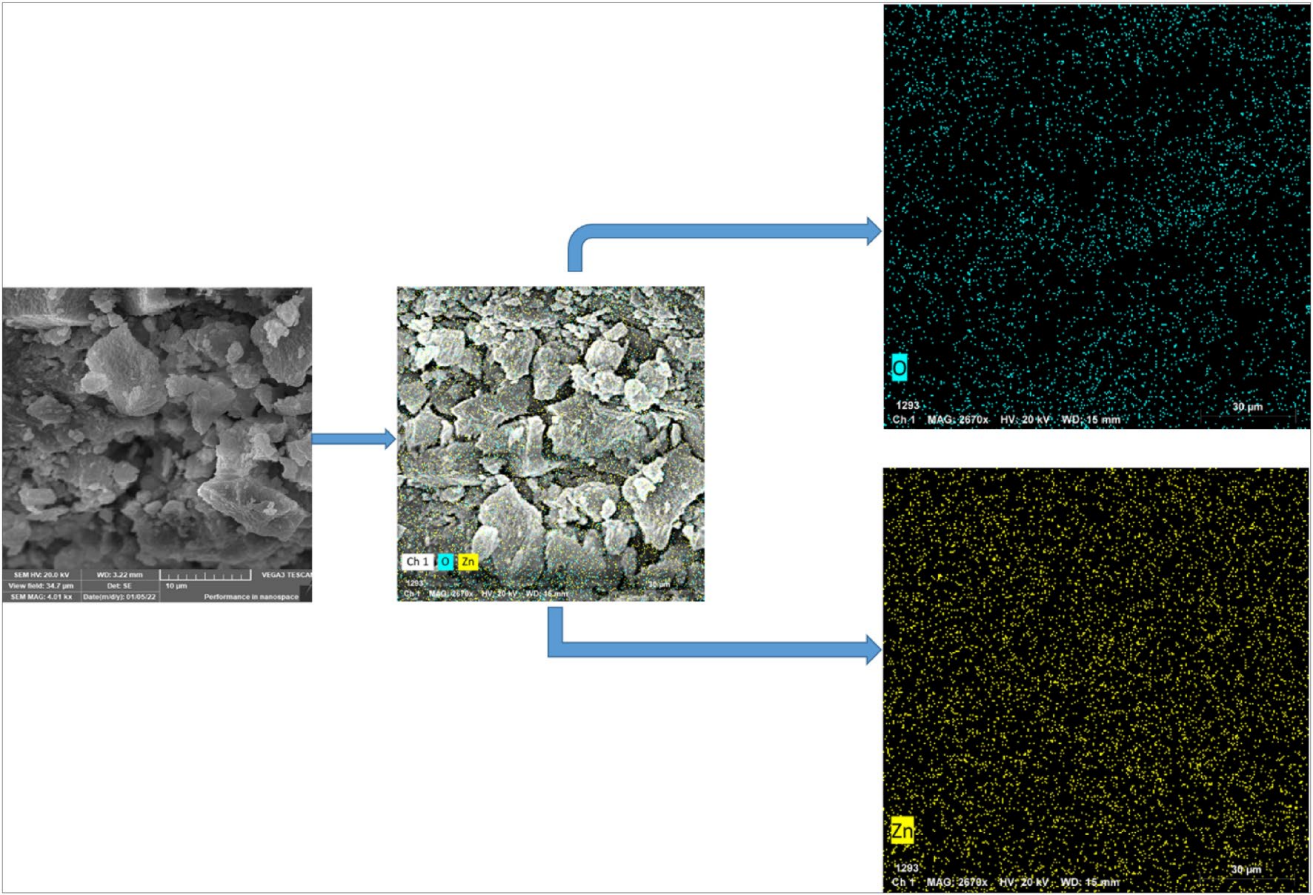
Scanning electron microscopic micrographs of bacterial synthesized ZnO-NPs.

**Figure 7:**
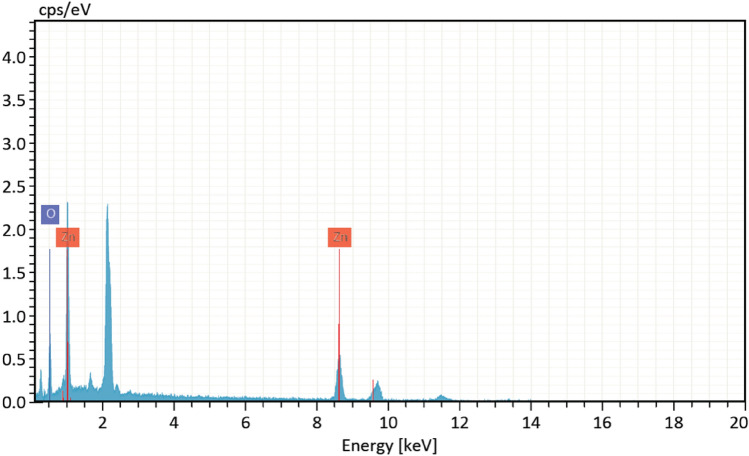
Elemental analyses of bacterial synthesized ZnO-NPs.

Figure 6 illustrates the graph of SEM microscopy, representing the formation of ZnO after treatment by the supernatant of the cultivated *Streptomyces pseudogriseolus*. The formation of oxides from Zn metals was confirmed by the elemental analyzing technique (EDX) as illustrated in Fig. 7. From these data, we can conclude the formation of zinc oxide by reducing agents found in the supernatant of *Streptomyces pseudogriseolus.* These investigations are also noted by other previous works^23^.

#### Cytotoxic evaluation of the actino-synthesized ZnO-NPs against BJ1, human normal fibroblast cell line

To evaluate the cytotoxicity of the produced ZnO-NPs, their effect on normal human epithelial cells was estimated. The samples were tested against the normal human epithelial cell line: BJ1 (normal Skin fibroblast) at concentrations ranging between 0.78 and 100 µg/mL using MTT assay. At the highest tested concentration (Table 2), the number of dead cells is less than half of the total initial cell count. The obtained results comply with the data reported by the US Food and Drug Administration that consider zinc compounds as safe (GRAS) substances, ZnO nanoparticles have biosafety, biocompatibility, and lack of toxicity even after daily use^13^. Also, ZnO-NPs are used as one of the ingredients of global sunscreen protection products.

**Table 2:** Cytotoxicity evaluation of ZnO-NPs using MTT assay.

Sample code	Percent of cell death at 100 ppm (%)
ZnO-NPs	47.3
DMSO	1
Negative control (deionized water)	0

## Characterization of ZnO-NPs-loaded polyester/cotton fabrics

### EDX analyses

**Figure 8:**
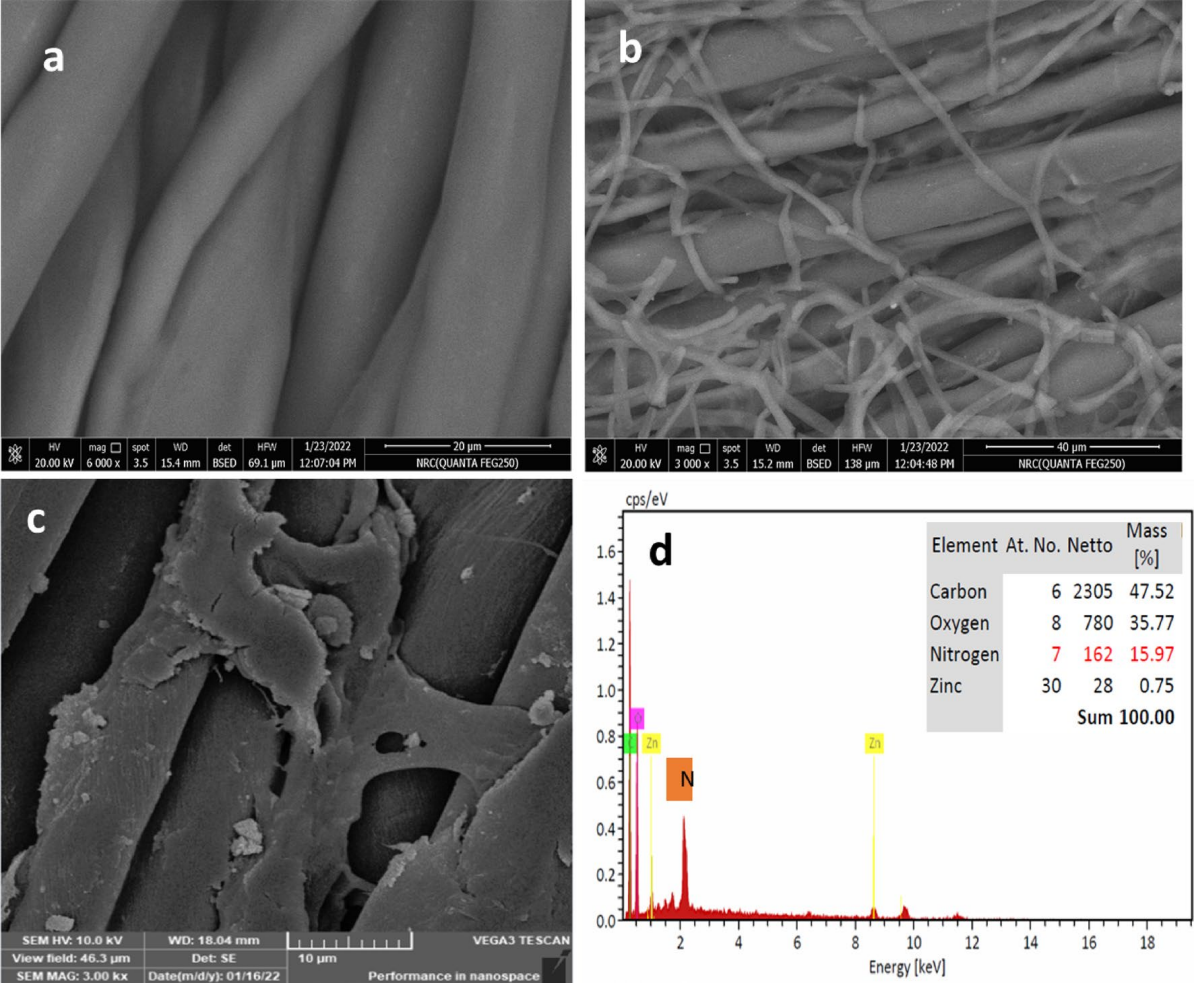
SEM and EDX images of (**a**) PET/C fibrous waste; (**b**) Activated PET/C fibrous waste by Cellulase; (**c**,**d**) PET/C activated fibrous waste loaded with ZnO-NPs(3000X).

EDX research verified the existence of ZnO-NPs on the PET/C fabric wastes' surface. Figure 8 displayed the EDX spectra of the fabrics loaded with ZnO-NPs after five washing cycles. It is significant to conclude that the deposited substance was composed of zinc and oxygen based on these spectra. It can be observed that ZnO remains on the fabric's surface even after five washing cycles, or twenty-five household washings. Higher Zn concentration on treated cloth wastes by Cellulase is also revealed by EDX testing (Zn atomic weight percentage was 0.75). This indicates that ZnO-NPs have adequate adherence to the activated fabric wastes.

#### Surface topography

SEM analysis for the samples was also conducted (Fig. 8) to examine the morphology of the modified polyester textiles loaded by ZnO-NPs. It showed the images of the activated and loaded fabrics with NPs followed by five washing cycles. Figure 10A shows that the surface of the parent PET/C blended fabric waste was clean and smooth. After treatment by Cellulase, a few damaged fibers appeared on the surfaces of PET/C, and the latter has gained a rough fabric Surface (Fig. 8b). A thinner, more homogeneous surface layer covers the activated polyester textiles and loaded with NPs (Fig. 8c and d); this clearly demonstrates a continuous deposited substance.

#### Antimicrobial assessment of NPs

Actinobacterial-ZnO-NPs were tested for their antimicrobial activity against Gram-positive, Gram-negative, and non-filamentous fungi (candida). By measuring the inhibitory zone width surrounding the sample in mm without taking into account the desk or well diameter, the activity by diffusion is measured. The data listed in Table 3 showed that high antibacterial activity was demonstrated by the synthesized ZnO-NPs against the specified harmful pathogens. The role of preparing ZnO-NPs by actinobacteria seems to be significant. The activated and loaded waste was also tested for its ability to inhibit the growth of Gram-positive, Gram-negative, and non-filamentous fungi. Also, the MIC was ranged between 50 and 70 µg/mL against the tested pathogens. These data demonstrate that all samples exhibited strong antibacterial activity against the three pathogens listed above following five washing cycles. Because the samples were repeatedly laundered in a launder-Ometer, the persistence of the activation of polyester fabrics with cellulase and loading with ZnO-NPs appears to be more important. This again verifies the feasibility of increasing the OH content in cotton fibers before its finishing with NPs. These results also show that, the activation of fibrous waste before loading with NPs enhancement the attachment of these NPs on the fiber surfaces. Also, activated fibrous waste and loaded with ZnO-NPs showed high antimicrobial activity and after washing 5 cycles all samples still provide antimicrobial activities. The antimicrobial activities can be explained by the fact that NPs loaded on the activated fibrous waste diffuse inside the microorganism’s cell and kill them.

**Table 3:** Antimicrobial activity of actinobacteria biosynthesized ZnO-NPs.

Samples	Inhibition zone diameter (mm) for polyester fabrics coated with biosynthesized zinc oxide nanoparticles						
	*B. c*	*L. m*	*E. f*	*E. c*	*Ps. a*	*S. t*	*C. a*
Amoxicillin	23	25	22	24	25	26	–
Nystatin	–	–	–	–	–	–	20
ZnO-NPs (100 µg/mL)	22	24	20	21	22	25	20
PET/C (1 cm)	00	00	00	00	00	00	00
PET/C + E (1 cm)	00	00	00	00	00	00	00
PET/C + E + ZnO-NPs after washing for 1 cycle (1 cm)	18	19	17	16	15	17	15
PET/C + E + ZnO-NPs after washing for 5 cycles (1 cm)	15	16	15	13	14	13	12

where *B. c.*, *Bacillus cereus*, *L. m.*, *Listeria monocytogenes*, *E. f.*, *Enterococcus faecalis*, *Ps. a.*, *Pseudomonas aeruginosa*, *S. t.*, *Salmonella typhi*, *E. c.*, *Escherichia coli* and *C. a.*, *Candida albicans*, the fiber samples washed for 1 and 5 cycles according to American Association of Textile Chemists and Colorists (AATCC) test method (61-1989), E = cellulases enzyme, PET/C = Polyester/Cotton.

#### Properties that protect against ultraviolet

Investigations were conducted into the impact of activating PET/C mix fabric waste with cellulase prior to loading it with ZnO-NPs on the effectiveness toward UV protection. The UPF values provided in Table 4 were used to quantify and express the rate of UV protection. The UPF factors for the parent waste cloth and the activated PET/C blend were found to be 11.2. Activation with Cellulase followed by loading with *Streptomyces pseudogriseolus* synthesized ZnO-NPs admission onto the above-stated fabrics led to a substantial increase in UPF factor to the level equivalent to UPF rating of 50+, which gives the excellent UV protection after 5 washing cycles. These findings suggest that polyester/cotton fabrics overloaded with ZnO-NPs after being activated with cellulase enzyme have outstanding laundry durability.

**Table 4:** Activity of PET/C blended waste fabrics as a UV protector.

Fabrics	UPF values after no of washing cycles:			
	1*		5*	
	UPF value	UPF** Rating	UPF value	UPF** rating
PET/C	10.5	Poor	–	–
PET/C + E	11.2	Poor	–	–
PET/C + E + ZnO-NPs	75.3	Excellent	59.6	Excellent

Where, *The fiber samples washed for 1 and 5 cycles according to American Association of Textile Chemists and Colorists (AATCC) test method (61-1989) and Australia (AS) / New Zealand (NAS) Standard No. 4399 (1996), E = cellulases enzyme, PET/C = Polyester/Cotton.

## Materials and methods

### Materials

Polyester-cotton blended (50/50 of PET/C) fabric waste was as a filament woven textile made from yarns of filament. The samples were collected from Misr Elamerya Co., Alex, Egypt. The textiles were worn at 80 °C/45 min with 2 g/L nonionic detergent solution, and then washed by tap water, squeezed, and dried in open air. Acid cellulase enzyme used in the current work was a multifunctional cellulases enzyme as formulations of Cellusoft® L (Novo Nordisk).

### Enzyme activation of fibrous wastes

**Figure 9:**
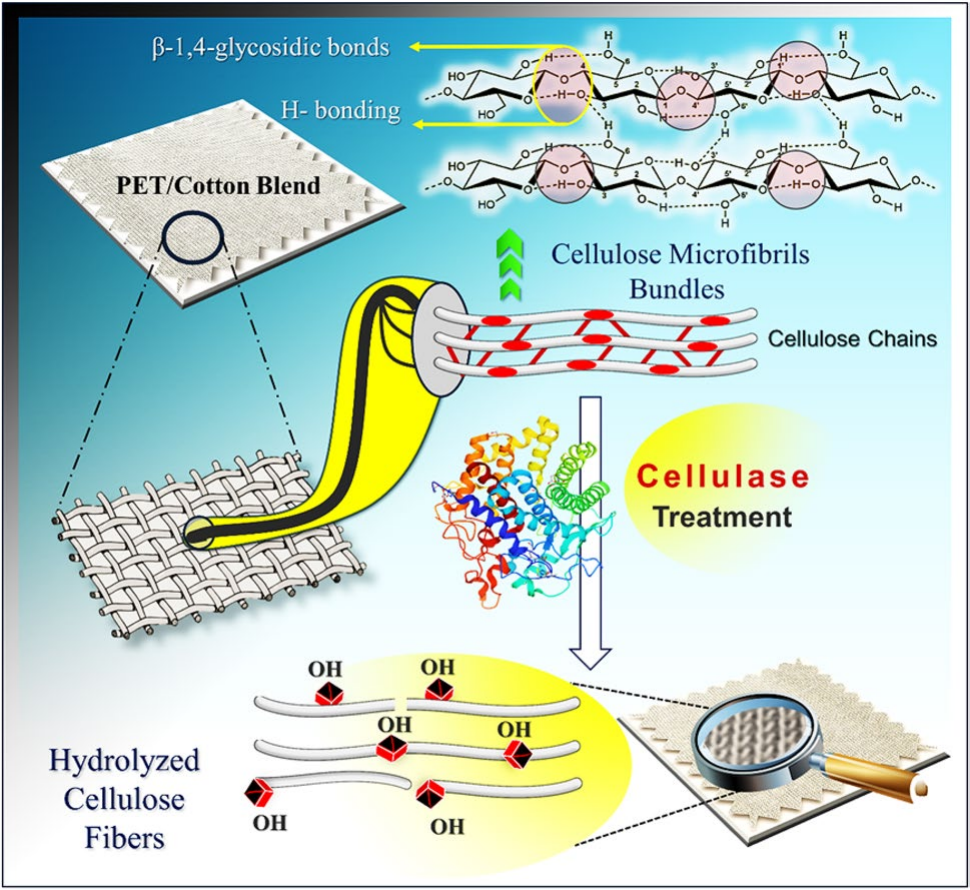
Schematic diagram for experimental representation for enzymatic surface activation of cotton blend substrate with cellulase enzyme.

To activate PET/C blended waste using Cellusoft® L enzyme, a high-temperature high-pressure laboratory dyeing machine was utilized. According to our previous study^11^, the appropriate enzyme amount was employed in bowls of stainless steel, and the collected waste samples were submerged in the enzyme solution with a pH of 5.0. The sealed bowls were then rotated in a closed bath containing ethylene glycol at 50°C, with a material-to-liquor ratio of 1:15. The bath temperature increased gradually at a rate of 5°C per minute until reaching 90 °C. After 24 h, the enzyme activity was inactivated by increasing the temperature to 90 °C, and the samples were taken out of the water bath and rinsed several times in hot and cold distilled water parallel. Finally, the treated samples were dried in the open air (Fig. 9).

The enzymatic-degradation ratio in the treated samples was estimated by evaluating the weight loss (WL) % of the fiber samples based on the following equation:

$$\text{WL }\left(\%\right)=\frac{W1-W2}{W1}\times 100$$

where W_1_ and W_2_ are the weights of the samples before and after enzymatic treatments, respectively.

### Microbial production of ZnO nanoparticles

#### Isolation and identification of Zn ion-resistant actinobacteria

Soil samples were collected from four locations in the Giza governorate. The samples were collected in clean plastic bags at a depth of 10–20 cm from the cultivation farms. Actinobacterial isolates were obtained and maintained on a starch-casein agar medium. Isolation procedures were conducted according to the methods described by Abo-Alkasem et al.^24^. Pure isolates were inoculated on agar slants of the starch-casein medium; colonies were labeled with a code referring to the source of the collection place. Eight isolates out of 64 isolates were found to be resistant to zinc ions supplemented to the starch-casein agar medium with a concentration of 0.1% as described by Darwesh et al.^11^. The isolates were subjected to morphological characterization based on the color of the sporulated aerial mycelium, substrate mycelium and the diffusible soluble pigments other than melanin. The growth profile and color observations were recorded and interpreted^25^. Also, the color was described according to the ISCC-NBS color chart^26^.

#### Screening for ZnO-NPs producing actinobacteria

The isolated actinobacteria were screened for their ability to produce ZnO-NPs. Each isolate was inoculated into ISP2 broth medium and incubated at 28°C for 3 days. After incubation, the bacterial biomass was removed by centrifugation at 4193 xg for 5 min, and the supernatant was used as a source of reduction system to produce zinc nano-form. A mixture of the supernatant and a solution of 1000 mg/L zinc acetate (1:1) was prepared and incubated overnight under shaking at 100 rpm. The mixture was centrifuged at 16,770 xg for 15 min and the precipitates were washed three times by deionized water and collected. The best producer isolate (NRC-MO23) for ZnO-NPs was used to produce zinc nanostructure based on its activity for producing reducing agents to convert Zn^2+^ to its nanostructures as determined by spectrophotometer absorption.

#### Identification of selected actinobacterial isolate for ZnO-NPs biosynthesis

The actinobacterial isolate (NRC-MO23) selected based on its efficiency in the biosynthesis of ZnO-NPs was subjected to morphological/microscopically characterize^27^ and molecular identification via determination of its 16s rRNA sequence. In brief, the genomic DNA was extracted from the most active isolate and the 16S rRNA gene was amplified by PCR using a Bio-Rad T100 thermal cycler (Bio-Rad Laboratories, CA, USA) as previously described^28^. The produced PCR fragments were purified using a Gel-PCR purification Kit (QIAquick, Qiagen, USA). The purified 16S rRNA fragments were examined by electrophoresis of agarose gel and visualized using UV-transilluminator^29^. Sequencing processes of the amplified 16S rRNA fragments were performed using a BigDyeR Terminator v3.1 Cycle Sequencing Kit (Applied Biosystems, Carlsbad, CA, USA) on an Applied Biosystems 3730xl DNA Analyzer. Similarities of the obtained nucleotide sequences with other known sequences were studied by comparisons with the National Center for Biotechnology Information (NCBI) database for reference and type strains using the BLASTN program (https://blast.ncbi.nlm.nih.gov/Blast.cgi). The phylogenetic tree based on partial 16S rRNA sequences was created using the neighbor-joining technique contained the Clustal X program and MEGA6 software. The obtained sequences were submitted to GenBank.

#### Extracellular production of ZnO-NPs using *Streptomyces pseudogriseolus*

Extracellular actino-synthesis of ZnO-NPs was performed using its aqueous cell-free supernatant of a three-day-old culture. *Streptomyces pseudogriseolus* was cultivated in a three-litter conical flask containing two liters of ISP2 broth medium and incubated at 28 °C for three days. Cell-free supernatant was obtained by filtering the *S. pseudogriseolus* culture through Whatman paper No. 1 and then centrifuged. The actinobacterial supernatant was combined with an equivalent volume of zinc acetate solution (0.5%), and the resultant mixture was stirred for a full night at 28 °C in dark conditions at 100 rpm. Centrifugation at 12,298 xg for 15 min was used to gather the precipitates. After that, they were washed twice with 100% ethanol after three times with deionized water. Finally, they were dried at 50 °C in an oven to achieve a constant weight. The spectrophotometer was calibrated to scan the precipitates at 350 nm in order to verify the production of ZnO-NPs. The dried and grounded ZnO-NPs were collected and subjected to further characterization.

### Structural characterization of the actino-synthesized ZnO-NPs

#### Spectroscopic investigation

UV–Vis spectroscopy was used to measure an aqueous solution of the prepared ZnO-NPs within the range of 200–800 nm using a spectrophotometer (UV–Vis, 1401 Shimadzu).

#### Particle size determination using TEM

The size and morphological shape of actino-synthesized ZnO-NPs were investigated by high-resolution transmission electron microscopy (HRTEM, JEOL 2100 Japan®). A thin film of the sample solution was placed onto the carbon-coated copper TEM grid and then dried and loaded into the specimen holder. After taking the HRTEM micrograph, the dimensions and formed shape were noted.

#### Functional group estimation using FTIR

Treating Zn solution with microbial culture filtrate is responsible for the reduction mechanism which converts the metal into nanoform and for the capping and active stabilization of the produced actino-synthesized ZnO-NPs. Thereby FTIR spectrophotometer was used to determine the functional groups of the bio-molecules responsible for exerting the stabilization and capping effect. One mg of ZnO-NPs was mixed with 2.5 mg of dry potassium bromide (KBr) in a mortar and grinded using a pestle. The obtained powder was placed in a 2 mm internal diameter micro cup and loaded onto the FTIR set at 26 ± 1 °C. The samples were scanned using infrared in the range of 4000: 400 cm^−1^ using an FTIR spectrometer (Agilent system Cary 630 FTIR model). The obtained spectral data were annotated to identify the functional groups present in the sample.

#### Crystalline structure characterization of actino-synthesized ZnO-NPs

XRD analysis was conducted using XRD-6000 series by Shimadzu apparatus. Nickel-filter and Cu-Kα X-ray targets were used on PAN analytical Xpert PRO Instruments, Holand. The parameters were adjusted at 2θ scan range (10–80), step side (0.02), scan rate (0.5 s), and the anode was made of copper.

#### Morphological (surface) and elemental composition investigation of ZnO-NPs

The topography and morphology of the actino-synthesized ZnO-NPs have been studied using a scanning electron microscope with energy-dispersive X-ray (SEM–EDX) (JEOL-Model JSM T20, Tokyo, Japan). After coating the tested powder by gold layer, the samples were analyzed.

#### Carboxylic content

The carboxylic content of parent and activated fibrous waste was tested based on the method designated by Darwesh and his co-workers^11^.

#### Cytotoxicity evaluation of the actino-synthesized ZnO-NPs on human normal fibroblast cell line (BJ1)

Cytotoxicity property test was conducted by evaluating the cell viability using the mitochondrial-dependent reduction of yellow MTT (3-(4,5-dimethylthiazol-2-yl)-2,5-diphenyl tetrazolium bromide) to purple formazan^30^. The cells were suspended in DMEM-F12 medium supplemented with 1% of an antibiotic mixture (10,000 U/mL Potassium Penicillin, 10,000 µg/mL Streptomycin Sulfate, and 25 µg/mL Amphotericin B), and 1% L-glutamine at 37 °C under 5% CO_2_. Cells were batch cultivated for ten days, then seeded at a concentration of 10 × 10^3^ cells/well of fresh growth medium in 96-well microtiter plates at 37 °C for 24 h under 5% CO_2_ using a water-jacketed Carbon dioxide incubator (Sheldon, TC2323, Cornelius, OR, USA). After aspirating the media and adding fresh medium (devoid of serum), the cells were cultured with varying concentrations of the sample, resulting in final concentrations (100–50–25–12.5–6.25–3.125–1.56 and 0.78 µg/mL), which was then compared with the negative control (the blank medium). After 48 h of incubation, the medium was aspirated, 40 µl MTT salt (2.5 μg/mL) was added to each well and incubated for further four hours at 37 °C under 5% CO_2_. To stop the reaction and dissolve the formed crystals, 200 μL of 10% sodium dodecyl sulphate (SDS) in deionized water was added to each well and incubated overnight at 37 °C. DOX was used as a positive control at 100 µg/mL giving 100% lethality under the same conditions^31^. Next, the absorbance was determined at 595 nm using a reference wavelength of 620 nm and a microplate multi-well reader (Bio-Rad Laboratories Inc., model 3350, Hercules, California, USA). Graph Pad Prism was used for statistical analysis (nonlinear regression curve fit approach). DMSO is the vehicle used for the dissolution of the formed ZnO-NPs and its final concentration in the cells was less than 0.2%. The percentage of change in viability was calculated according to the formula:

$$\left( {\text{Reading of extract}}/{\text{Reading of negative control}} \right) - {1}) \times 100.$$

#### Fabrication of the functionalized PET/C fibrous waste

**Figure 10:**
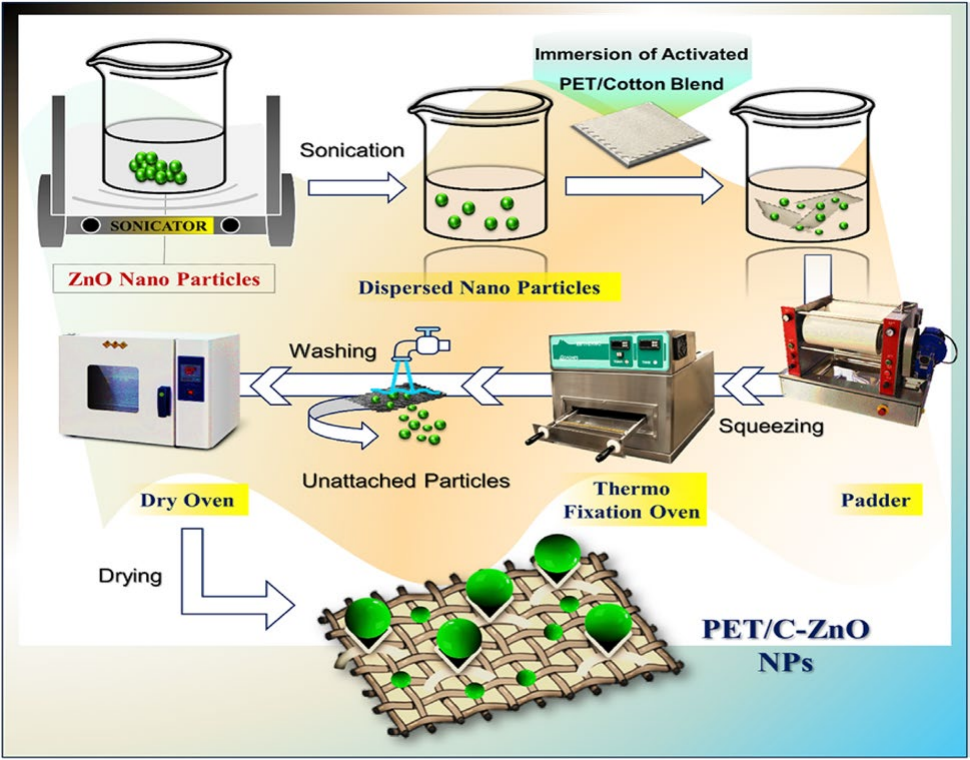
Experimental representation for surface decoration of activated cotton blend substrate with biosynthesized ZnO-NPs.

The activated PET/C blended waste fabrics by cellulases were immersed in the ZnO-NPs aqueous dispersion (sonicated for 30 min). After that, a padder was used to squeeze the samples so that 60% (w/w) of the solution was removed and then dried in the open air for 24 h. Finally, cured in a thermo-fixation oven at 150°C for 15 min. In order to evaluate the NPs adhesion to the fibrous waste, the normal procedure (AATCC, 61-1989) was followed to wash the treated samples five times (Fig. 10).

#### Antimicrobial assessment

The actinobacterial-synthesized ZnO-NPs were tested as antimicrobial material against various reference microbial pathogens like *Bacillus cereus* ATCC-12228, *Listeria monocytogenes* ATCC-35152, *Enterococcus faecalis* ATCC-29212, *Pseudomonas aeruginosa*, *Salmonella typhi*, *Escherichia coli* ATCC-25922 and *Candida albicans* ATCC-10231^32^. The well diffusion agar method, as previously designated by Eweys et al.^33^ was applied to test the antimicrobial action. ZnO-NPs were evaluated and compared with Nystatin, an anti-candidal reference, and Amoxicillin, an antibacterial reference, at a concentration of 200 µg/mL of each. Each sample was tested three times, and the average values were used to describe the findings. Additionally, the minimum inhibitory concentration (MIC) was established to define the suitable concentration needed to upload onto fibers. On the other way, the antimicrobial activity of the activated PET/C blended fabric waste loaded with the produced ZnO-NPs was measured utilizing the disc diffusion method^34^. This technique measured the antibacterial efficacy via diffusion by measuring the zone of growth inhibition surrounding the sample (in mm).

#### Ultraviolet protection

Ultraviolet protection factor (UPF) was measured using UV-Shimadzu 3101 P C -Spectrophotometer. It is a double-beam direct ratio measuring system. The UPF factor was calculated using the procedure outlined in the Australian/New Zealand Standard AS/NZS 4399: 1996^35^.

## Conclusions

The current work presents a green technique to increase actino-synthesized ZnO-NPs' capacity to bind onto waste PET/C mix fabrics. The pad-dry-cure approach is used to load polyester/cotton fabrics with ZnO-NPs after the biological activation procedure by Cellusoft® L (Novo Nordisk) is applied. The superior isolate for ZnO-NPs biosynthesis was identified as *Streptomyces pseudogriseolus* and submitted in Genebank (OR574241). The biosynthesized ZnO-NPs and modified fabrics were characterized by SEM, EDX, and FTIR spectroscopy. Also, the visual vision, cytotoxicity, and bioactivity of prepared NPs were determined. When compared to parent fabrics, PET/C fabrics treated with an enzyme prior to being loaded with ZnO-NPs showed superior antibacterial and UV protection qualities. Overall, the data gathered for this study point to the potential use of the biological surface activation technique to attach the actinobacteria-produced ZnO-NPs to polyester/cotton textiles. This suggested approach is paving the way for using the prepared smart fibrous waste in high-tech applications such as wastewater treatment, packaging and electro-bio sensors.

### Supplementary Information

Supplementary Information.

## Data Availability

The data that support the findings of this study are available from the corresponding author upon reasonable request.
