# Supplementary material for: Circular economy reinforcement through molecular fabrication of textile wastes with microbial synthesized ZnO nanoparticles to have multifunctional properties
Source: Sci Rep. 2024 Jul 19;14:16660. doi: 10.1038/s41598-024-66430-1 (PMC11271589; doi:10.1038/s41598-024-66430-1)
Supplement: Supplementary file 1 — Supplementary Information. [file 41598_2024_66430_MOESM1_ESM.docx]

Supplementary data

1. **Media used in this study**
   1. **Basal mineral salts agar (g/L) (Shirling and Gottlieb, 1966)**

(NH_4_)_2_SO_4_, 2.64; KH_2_PO_4_, 2.38; K_2_HPO_4_.3H_2_O, 5.65; MgSO_4_. 7H_2_O, 1.00; Pridham and Gottlieb trace elements solution, 1ml; Agar 15.0; Distilled water up to 1000 ml. The pH of the medium was adjusted at pH 7.0 before sterilization. The composition of the trace salt solution according to (Pridham and Gottlieb, 1948) was as follows (g/1; w/v): CuSO_4_.5H_2_O, 0.64; FeSO_4_.7H_2_O, 0.11; MnCl_2_.4H_2_O, 0.79; ZnSO_4_.7H_2_O, 0.15, Distilled water up to 1000 ml.

- 1. **Casein-starch agar medium (g/L) (Kuster and Williams, 1964)**

Soluble starch, 10.0; Casein, 0.3; KNO_3_, 2.0; NaCl, 0.5; K_2_HPO_4_, 2.0; MgSO_4_.7H_2_O, 0.5; CaCO_3_, 2.0; FeSO_4_.7H_2_O, 0.01; Agar, 20.0; Distilled water up to 1000 ml and pH value of the medium was adjusted at 7.2 before sterilization.

- 1. **Czapek's agar medium (g/L) (Shirling and Gottlieb, 1966)**

Sucrose, 30.0; NaNO_3_, 2.0; K_2_HPO_4_, 1.0; KH_2_PO_4_, 0.5; KCl, 0.5; FeSO_4_.7H_2_O, 0.01; Agar, 20.0; Distilled water up to 1000 ml. The pH was adjusted at 7.5 before sterilization.

- 1. **Czapek's yeast extract casamino acid (CYC) agar (g/L) (Lacey and Cross, 1989)**

Czapek-Dox agar powder, 33.4; yeast extract, 2.0; Vitamin free casamino acids, 6.0; Distilled water up to 1000 ml, the pH of the medium was adjusted at 7.2 before sterilization.

- 1. **Difco™ Marine Agar (g/L) (ZoBell, 1941)**

Peptone, 5.0; Yeast extract, 1.0; (C_6_H_5_)FeO_7_, 0.1; NaCl, 19.45; MgCl_2_, 8.8; Na_2_SO_4_, 3.24; CaCl_2_, 1.8 ; KCl, 0.55; Na_2_CO_3_, 0.16; KBr, 0.08; SrCl_2_, 0.034; Boric Acid, 0.022; Na_2_SiO_3_, 0.004; NaF, 0.0024; NH_4_NO_3_, 0.0016; Na_2_HPO_4_, 0.008; Agar, 15.0; Distilled water up to 1000 ml. The pH of the medium was adjusted at pH 7.6 before sterilization.

- 1. **Glycerol-asparagine agar (ISP-5) (g/L) (Pridham and Lyons, 1961)**

Glycerol, 10.0; L-asparagine (anhydrous), 10.0; K_2_HPO_4_, 1.0; Trace salt solution, 0.1 ml; Agar, 20.0; Distilled water up to 1000 ml. The pH of the medium was adjusted at 7.3 before sterilization.

- 1. **Inorganic salts starch agar medium (ISP-4) (g/L) (Kuster, 1959)**

Solution I: Soluble starch, 10.0; Distilled water, 500 ml. A paste of starch with a small amount of cold distilled water was prepared and then completed to 500 ml with distilled water. Solution II: K_2_HPO_4_ (anhydrous), 1.0; MgSO_4_.7H_2_O, 1.0; NaCl, 1.0; CaCO_3_.H_2_O, 2.0; Trace salt solution, 1.0 ml; Agar, 20.0; Distilled water, 500 ml. pH adjusted to 7-7.4 before sterilization.

- 1. **Modified Bennett's agar (g/L) (Williams et al., 1983)**

Beef extract, 1.0; Glycerol, 10.0; Enzymatic digest of casein, 2.0; Yeast extract, 1.0; Agar 15.0; Distilled water up to 1000 ml. The pH of the medium was adjusted at pH 7.3 before sterilization.

- 1. **Non-nutrient agar (NNA) (g/L) (Page, 1976)**

Na_2_HPO_4_, 0.142; KH_2_PO_4_, 0.136; NaCl, 0.12; MgSO_4_.7H_2_O, 0.004; CaCl_2_, 0.004; Agar, 15.0; Distilled water up to 1000 ml. The pH of the medium was adjusted at pH 7.0 before sterilization.

- 1. **Nutrient agar (NA) (g/L) (MacFaddin, 1985)**

Beef extract, 3.0; Peptone, 5.0; Sodium chloride, 5.0; Agar, 20.0; Distilled water 1000 ml. The pH was adjusted at 6.8 before sterilization.

- 1. **Nutrient broth (g/L) (MacFaddin, 1985)**

Beef extract, 3.0; Peptone, 5.0; Sodium chloride, 5.0; Distilled water 1000 ml. The pH was adjusted at 8.5 before sterilization.

- 1. **Oatmeal agar (ISP-3) (g/L) (Kuster, 1959)**

Oatmeal, 20.0; Trace salt solution, 0.1; Agar, 20.0; Distilled water up to 500 ml. The oatmeal was boiled in 1000 ml distilled water for 20 min, filtered through cheese cloth and distilled water was added to restore the volume of the filtrate to 1000 ml. The pH was adjusted at 7.2 and then agar was added. The composition of the trace salt solution according to (Pridham and Gottlieb, 1948) was as follows (g/1; w/v): CuSO_4_.5H_2_O, 0.64; FeSO_4_.7H_2_O, 0.11; MnCl_2_.4H_2_O, 0.79; ZnSO_4_.7H_2_O, 0.15, Distilled water up to 1000 ml.

- 1. **Peptone yeast extract-iron agar medium (ISP-6) (g/L) (Shirling and Gottlieb, 1966)**

Bacto-peptone, 15.0; Proteose peptone (Difco), 5.0; Ferric ammonium citrate, 0.5; K_2_HPO_4_, 1.0; Sodium thiosulfate, 0.08; Bacto-yeast extract, 1.0; Agar, 20.0; Distilled water up to 1000 ml. The pH value was adjusted at 7-7.2 before autoclaving.

- 1. **Plate count agar (PCA) medium (g/L) (Reasoner and Geldreich, 1985)**

Tryptone, 5.0; Yeast extract, 2.5; Glucose, 1.0; Agar, 12.0; Distilled water up to 1000 ml. The pH of the medium was adjusted at pH 7.0 before sterilization.

- 1. **Potato dextrose agar (PDA) (g\l) (MacFaddin, 1985)**

Potato Infusion from 200 g; Dextrose, 20 g; Agar 15 g; Distilled water up to 1000 ml, the pH of the medium was adjusted at 6.8 before sterilization

- 1. **Starch-nitrate agar medium (SNA) (g/L) (Tadashi, 1975)**

Soluble starch, 20.0; NaNO_3_, 2.0; K_2_HPO_4_, 1.0; KCl, 0.5; MgSO_4_.7H_2_O, 0.5; CaCO_3_, 2.0; Agar, 20.0; Distilled water up to 1000 ml. The pH was adjusted at 7.0 before sterilization.

- 1. **Tryptone-yeast extract broth medium (ISP-1) (g/L) (Pridham and Gottlieb, 1948)**

Bacto-tryptone, 5.0; Bacto-yeast extract, 3.0; Distilled water up to1000 ml. The pH of the medium was adjusted at 7-7.2 before sterilization.

- 1. **Tyrosine agar (ISP-7) (g/L) (Shirling and Gottlieb, 1966)**

L-tyrosine (Difco), 5.0; Glycerol, 15.0; L-asparagine (Difco), 1.0; K_2_HPO_4_, 0.5; MgSO_4_.7H_2_O, 0.5; NaCl, 0.5; FeSO_4_.5H_2_O, 0.01; Trace salt solution, 1.0; Agar, 20.0; Distilled water up to 1000 ml. pH was adjusted at 7.2-7.4 before sterilization.

- 1. **Yeast extract-malt extract agar medium (ISP-2) (g/L) (Pridham et al., 1958)**

Yeast extract, 4.0; Malt extract, 10.0; Glucose, 4.0; Agar, 20.0; Distilled water, up to 1000 ml. The pH was adjusted at pH 7.2 -7.4 before sterilization.

**Results**

**Cultural characteristics of Actinobacterial isolates on different ISP media:**

**Table S1.** Cultural characteristics of Actinobacterial isolate NRC-MO10 on different media

| **Soluble pigment** | **Color of the substrate mycelium** | **Color of the aerial mycelium** | **Growth** | **Nutrient medium** |
| --- | --- | --- | --- | --- |
| Yellow | Yellow | Green | Good | Yeast – malt extract agar (ISP 2) |
| Yellow | Green | Green | Good | Oat meal agar (ISP 3) |
| Yellow | Yellow | Light-Green | Good | Inorganic-trace salt- starch agar (ISP 4) |
| None | Pale-Gray | Green | Good | Glycerol asparagine agar (ISP 5) |
| Yellow | Dark-Brown | Brown | Good | Peptone yeast extract iron agar (ISP 6) |
| Pale-Yellow | Brown | Pale-Green | Good | Tyrosine agar (ISP 7) |
| Yellow | Brown | Green | Good | Starch-Casein agar |
| Yellow | Pale-Yellow | Pale-Gray | Good | Nutrient agar |

**Table S2.** Cultural characteristics of Actinobacterial isolate NRC-MO19 on different media

| **Soluble pigment** | **Color of the substrate mycelium** | **Color of the aerial mycelium** | **Growth** | **Nutrient medium** |
| --- | --- | --- | --- | --- |
| Brown | Dark-Brown | Beige | Good | Yeast – malt extract agar (ISP 2) |
| Brown | Brown | Gray | Good | Oat meal agar (ISP 3) |
| Pale-Yellow | Orange | Yellow | Good | Inorganic-trace salt- starch agar (ISP 4) |
| No growth | No growth | No growth | No growth | Glycerol asparagine agar (ISP 5) |
| Dark-Brown | Dark-Brown | Gray | Good | Peptone yeast extract iron agar (ISP 6) |
| None | Beige | Beige | Good | Tyrosine agar (ISP 7) |
| Light-Brown | Brown | Beige | Good | Starch-casein agar |
| Light-Brown | Brown | Brown | Good | Nutrient agar |

**Table S3.** Cultural characteristics of Actinobacterial isolate NRC-MO21 on different media

| **Soluble pigment** | **Color of the substrate mycelium** | **Color of the aerial mycelium** | **Growth** | **Nutrient medium** |
| --- | --- | --- | --- | --- |
| Brown | Brown) | Creamy | Good | Yeast – malt extract agar (ISP 2) |
| None | Light-Yellow | Beige | Good | Oat meal agar (ISP 3) |
| None | Beige | Beige | Good | Inorganic-trace salt- starch agar (ISP 4) |
| None | Creamy | Hygroscopic | Week | Glycerol asparagine agar (ISP 5) |
| None | Creamy | Hygroscopic | Weak | Peptone yeast extract iron agar (ISP 6) |
| None | Light-Yellow | Gray | Good | Tyrosine agar (ISP 7) |
| None | Beige | Creamy | Good | Starch-casein agar |
| None | Light-Yellow | Hygroscopic | Week | Nutrient agar |

**Table S4.** Cultural characteristics of Actinobacterial isolate NRC-MO23 on different media

| **Soluble pigment** | **Color of the substrate mycelium** | **Color of the aerial mycelium** | **Growth** | **Nutrient medium** |
| --- | --- | --- | --- | --- |
| Brown | Brown | Pale-Gray | Good | Yeast – malt extract agar (ISP 2) |
| Brown | Brown | Gray | Good | Oat meal agar (ISP 3) |
| Pale-Brown | Beige | Creamy | Good | Inorganic-trace salt- starch agar (ISP 4) |
| None | Creamy | Hygroscopic | Week | Glycerol asparagine agar (ISP 5) |
| Brown | Brown | Gray | Good | Peptone yeast extract iron agar (ISP 6) |
| None | Brown | Creamy to White | Good | Tyrosine agar (ISP 7) |
| Light-Brown | Beige | Gray | Good | Starch-casein agar |
| Light-Brown | Brown | Pale-Gray | Good | Nutrient agar |

**Table S5.** Cultural characteristics of Actinobacterial isolate NRC-MO31 on different media

| **Soluble pigment** | **Color of the substrate mycelium** | **Color of the aerial mycelium** | **Growth** | **Nutrient medium** |
| --- | --- | --- | --- | --- |
| Beige | Brown | Beige | Good | Yeast – malt extract agar (ISP 2) |
| Beige | Brown | Beige | Good | Oat meal agar (ISP 3) |
| No growth | No growth | No growth | No growth | Inorganic-trace salt- starch agar (ISP 4) |
| No growth | No growth | No growth | No growth | Glycerol asparagine agar (ISP 5) |
| Light-Brown | Brown | Beige | Good | Peptone yeast extract iron agar (ISP 6) |
| Light-Brown | Brown | Beige | Good | Tyrosine agar (ISP 7) |
| Light-Brown | Brown | Beige | Good | Starch-Casein agar |
| Light-Brown | Brown | Beige | Good | Nutrient agar |

**Table S6.** Cultural characteristics of Actinobacterial isolate NRC-MO40 on different media

| **Soluble pigment** | **Color of the substrate mycelium** | **Color of the aerial mycelium** | **Growth** | **Nutrient medium** |
| --- | --- | --- | --- | --- |
| None | Light Brown | Dark green | Good | Yeast – malt extract agar (ISP 2) |
| None | Yellowish | Green | Good | Oat meal agar (ISP 3) |
| None | Light Brown | Beige | Good | Inorganic-trace salt- starch agar (ISP 4) |
| None | White | Green | Good | Glycerol asparagine agar (ISP 5) |
| None | Pale-Brown | Pale-Brown | Good | Peptone yeast extract iron agar (ISP 6) |
| None | Light Brown | Gray | Good | Tyrosine agar (ISP 7) |
| Light-yellow | Yellow | Green | Good | Starch-casein agar |
| Light-yellow | Pale-Yellow | Pale-Gray | Good | Nutrient agar |

**Table S7.** Cultural characteristics of Actinobacterial isolate NRC-MO52 on different media

| **Soluble pigment** | **Color of the substrate mycelium** | **Color of the aerial mycelium** | **Growth** | **Nutrient medium** |
| --- | --- | --- | --- | --- |
| None | Light-Brown | Beige | Good | Yeast – malt extract agar (ISP 2) |
| None | Light-Yellow | Beige | Good | Oat meal agar (ISP 3) |
| None | Beige | Gray | Good | Inorganic-trace salt- starch agar (ISP 4) |
| None | Creamy | Hygroscopic | Week | Glycerol asparagine agar (ISP 5) |
| None | Light-Yellow | Beige | Good | Peptone yeast extract iron agar (ISP 6) |
| None | Yellow | Beige | Good | Tyrosine agar (ISP 7) |
| None | Beige | Beige | Good | Starch-Casein agar |
| None | Pale-Yellow | Beige | Good | Nutrient agar |

**Table S8.** Cultural characteristics of Actinobacterial isolate NRC-MO62 on different media

| **Soluble pigment** | **Color of the substrate mycelium** | **Color of the aerial mycelium** | **Growth** | **Nutrient medium** |
| --- | --- | --- | --- | --- |
| None | Brown | Pale-Gray | Good | Yeast – malt extract agar (ISP 2) |
| None | Beige | Gray | Good | Inorganic-trace salt- starch agar (ISP 4) |
| None | Creamy | Hygroscopic | Week | Glycerol asparagine agar (ISP 5) |
| None | Light-Brown | Beige | Good | Peptone yeast extract iron agar (ISP 6) |
| None | Gray | Beige | Good | Tyrosine agar (ISP 7) |
| None | Pale-Gray | Gray | Good | Starch-casein agar |
| None | Creamy | Gray | Good | Nutrient agar |


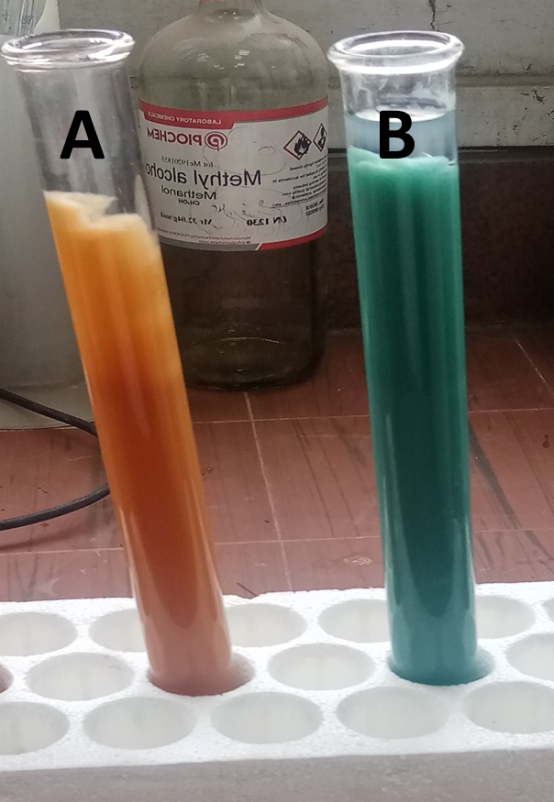


**Fig. S1.** Visual observation for formation of ZnO-NPs
